# Daytime Exposure to Blue-Enriched Light Counters the Effects of Sleep Restriction on Cortisol, Testosterone, Alpha-Amylase and Executive Processes

**DOI:** 10.3389/fnins.2019.01366

**Published:** 2020-01-08

**Authors:** Brice Faraut, Thomas Andrillon, Catherine Drogou, Caroline Gauriau, Alexandre Dubois, Aurélie Servonnet, Pascal Van Beers, Mathias Guillard, Danielle Gomez-Merino, Fabien Sauvet, Mounir Chennaoui, Damien Léger

**Affiliations:** ^1^EA 7330 VIFASOM (Vigilance Fatigue Sommeil et Santé Publique), Université de Paris, Paris, France; ^2^Consultation de Pathologie Professionnelle Sommeil Vigilance et Travail, Centre du Sommeil et de la Vigilance, Hôtel-Dieu, APHP-5, Paris, France; ^3^School of Psychological Sciences, Turner Institute for Brain and Mental Health, Monash University, Melbourne, VIC, Australia; ^4^Unité Fatigue et Vigilance, IRBA - Institut de Recherche Biomédicale des Armées, Brétigny-sur-Orge, France

**Keywords:** blue light, sleep restriction, stress, androgen, memory, attention

## Abstract

Sleep debt is becoming a better acknowledged cause of physiological stress and neurobehavioral deficits with major public-health concerns. We investigated whether exposure to blue light during daytime could be an efficient countermeasure to limit sleep restriction’s impact on relevant behavioral (stress, sleepiness, sustained attention, and memory performance) and physiological (saliva cortisol, testosterone, and alpha-amylase) markers. Our semi-ecological, crossover, randomized design included 17 young men that underwent two sleep-restricted nights (3 h each) followed or not by blue light exposure (30-min-long sessions at 100 lux repeated four times throughout the day). Behavioral and physiological measurements were performed in the lab but outside these periods the participants kept following their usual routine. After sleep restriction, morning cortisol and testosterone, and afternoon alpha-amylase levels decreased. In parallel, subjective ratings of stress and sleepiness increased while performance on the sustained attention and memory tasks deteriorated. In contrast, after periods of blue light exposure, all these parameters were largely restored to baseline levels, despite an identical sleep restriction procedure, although this restorative effect was reduced for the memory task. Our findings suggest that even short exposure to blue light could trigger persistent beneficial effects throughout the day and could be potentially efficient in real-life settings.

## Introduction

Reduced sleep [e.g., total sleep time (TST) less than 6 h per 24-h] has been reported in 20–30% of working adults (Léger et al., 2011; Ryu et al., 2011). Chronic sleep debt can lead to neurobehavioral deficits and physiological stress (e.g., sleepiness-related accidents cardiovascular risk and chronic diseases) and has become a major public health concern (Meerlo et al., 2008; Banks et al., 2010; Léger et al., 2011, 2014; Faraut et al., 2012; Gonçalves et al., 2015). The activity and reactivity of stress and immune systems depend on numerous environmental and individual factors, including the level of sleep debt (Besedovsky et al., 2012; Minkel et al., 2014). To limit the effects of this chronic sleep deprivation, napping (which can act as a stress-inhibiting factor) is emerging as one of the more obvious and efficient physiological countermeasures, but napping is not always transferable to the workplace (Faraut et al., 2015, 2017; Besedovsky et al., 2019). Light exposure, aside from napping or in combination, could represent an alternative or complementary countermeasure and deserves to be further investigated in the context of sleep restriction.

Indeed, in most species, including humans, light exposure participates in resynchronizing the biological clock by suppressing melatonin secretion during the biological day. Artificial light emitting in the blue short-wavelength domain of the visible spectrum (446–483 nm) has a direct effect on melatonin secretion, alertness levels, and cognitive performances, and this effect is even larger than white light due to the sensitivity within the blue-light spectrum of the retinal receptors involved (Lockley et al., 2006; Revell et al., 2006; Cajochen, 2007). Light exposure also impacts the release of cortisol from the hypothalamic-pituitary-adrenal (HPA) axis and catecholamines from the sympathetic nervous system (SNS; Leproult et al., 2001; Ishida et al., 2005).

Strikingly, the response to blue light exposure of salivary alpha-amylase (released by salivary glands that are innervated by the SNS), is largely unknown. Alpha-amylase is both an antimicrobial peptide with a key role in mucosal immunity and a surrogate of catecholamine changes in the blood, considered as a reliable stress marker of SNS activity (Nater and Rohleder, 2009). Two additional salivary markers reported to be sensitive to sleep deprivation and stress system activities, immunoglobulin A (IgA, which serves as a first line of immune defense against microbial infection) and testosterone (a gonadal steroid hormone involved in the development and maintenance of the male reproductive system, in muscle protein synthesis and vigor) were here assessed (Leproult and Van Cauter, 2011; Toufexis et al., 2014; Wada et al., 2017).

Sleep restriction also impacts behavioral performance, which has been extensively studied through simple cognitive tasks assessing individuals’ ability to respond to unpredictable stimuli or more complex paradigms involving memory or executive control (Banks and Dinges, 2007; Banks et al., 2010). Both physiological and cognitive consequences of sleep restriction could be interrelated, as physiological stress can impact behavioral performance (Meerlo et al., 2008). Accordingly, previous studies have shown a positive effect of blue light on some aspects of behavioral performance, but results for executive performance or working memory tasks are not always concordant and seems modulated by time of day and sleep pressure (Lockley et al., 2006; Cajochen, 2007; Gaggioni et al., 2014).

To better understand the effects of blue light exposure, we investigated whether repeated exposure to blue light in healthy subjects after two nights of restricted sleep could influence physiological (cortisol, alpha-amylase, testosterone, and IgA) and cognitive (subjective stress, sleepiness, mood and executive, and memory performances) markers of sleep debt. Importantly, our study design was performed in a semi-ecological protocol (behavioral and physiological measurements were performed in the laboratory but outside these periods the participants kept following their usual routine outside the laboratory) with 30 min of blue light repeatedly administered across the daytime period. This semi-ecological paradigm was designed to be as close as possible to what sleep-deprived adults experience on a daily basis and to warrant the transfer of our results to real-life settings.

## Materials and Methods

### Ethics Statement

The study was conducted according to French regulations on human research including agreements from the Hôtel-Dieu Hospital Ethics Committee (CPP Ile de France 1 – N° 2014-sept.-13690), with signed consent from participants who received financial compensation. Our protocol was conducted in accordance with the 2016 version of the Declaration of Helsinki and the ICH guidelines for Good Clinical Practice.

### Participants

Seventeen healthy men aged 19–33 years old [mean ± Standard-Error of the Mean (SEM) 23.9 ± 3.9 years] participated in the experiment.

#### Inclusion Criteria

• Healthy male volunteers between 18–35 years old;
• Weight proportional to height (BMI between 19–25);
• Regular nighttime sleep of 7–8 h with an intermediate chronotype as indicated by sleep and chronotype questionnaires (Pittsburgh Sleep Quality Index, Epworth Sleepiness Scale, Morningness–Eveningness Questionnaire; Horne and Ostberg, 1976; Buysse et al., 1989; Johns, 1991);
• Not a regular napper; non-smoker; no drug use. Each participant was in good health as reported by a preliminary medical examination, with no progressive ocular pathology (i.e., glaucoma, conjunctivitis or retinal degeneration) and no depression, anxiety or emotional distress as detected using the Hospital Anxiety and Depression Scale (Zigmond and Snaith, 1983).

#### Exclusion Criteria

• Subjects who participated in any trial within the 3 months preceding the study;
• Any drug treatment; renal or hepatic insufficiency;
• Any neurological or psychiatric disease; cardiac, gastro-intestinal, endocrinal or pulmonary or other chronic diseases; progressive ocular pathology; sleep complaints; extreme chronotype;
• Short sleeper; smoker; regular napper; shift or night worker;
• Any drug, alcohol, or caffeine abuse.

#### Recruitment

participants were recruited by advertising the study at the hospital and on the university campus. All participants were volunteers and gave their informed written consent. Due to technical issues, only part of the data was exploitable for subject 16.

### Experimental Design

**FIGURE 1:**
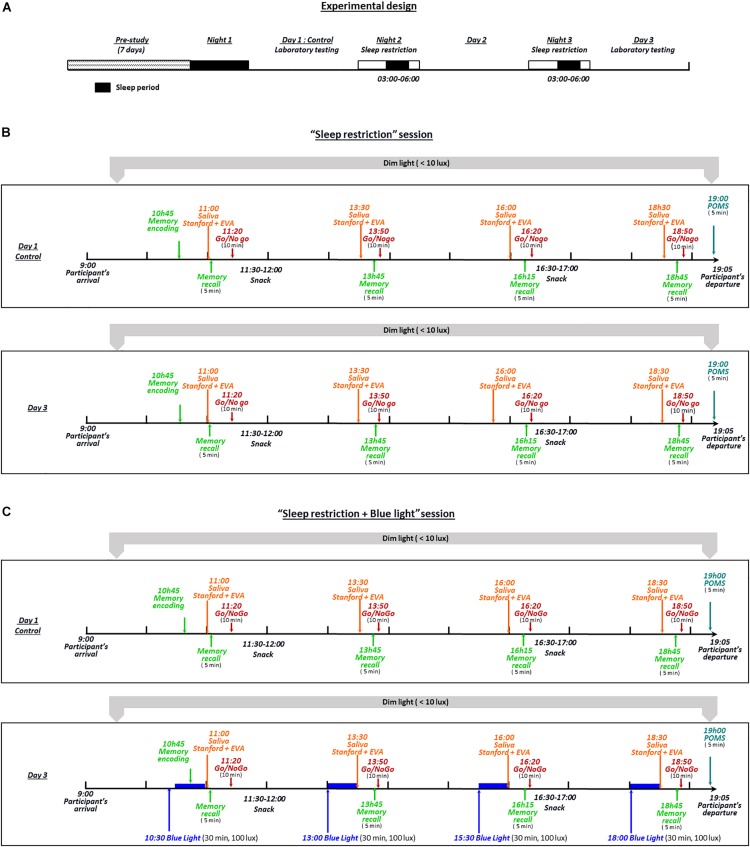
The experimental design included two 3-day sessions **(B,C)** performed by all participants in random order. During these two 3-day sessions, the participants were instructed to restrict their sleep time to 3 h for two consecutive nights (in bed from 03:00 to 06:00) **(A)**. During the “sleep restriction” session **(B)**, the participants continued their normal routine outside the laboratory, under remote supervision by the investigators. After the second sleep-restricted night, participants returned to the laboratory on the morning of day 3 and their actigraphy recordings were immediately analyzed to check their compliance with the imposed sleep-wake schedule. During day 1 (after habitual sleep, control condition) and day 3 of each session, the participants remained from 09:00 to 19:05 in the laboratory under dim light (luminance level of ≤10 lux) and continuous supervision by the investigators. In the “sleep restriction + blue light” session **(C)**, the same participants repeated the protocol described above, with the addition of four sessions of 30 min of blue light exposure (100 lux at eye level at 10:30, 13:00, 15:30, and 18:00) the day after the second sleep-restricted night.

For our study to be as close as possible to real-life settings, we designed a semi-ecological protocol as follows. Despite the fact that all biological samplings and measurements were performed in the laboratory, participants were allowed to continue with their normal routines outside of these testing periods. The experimental design included two 3-day sessions that the participants performed in random order (Figure 1). The protocol was performed at a period of low natural light in Paris (France) from the end of October to January. Prior to their admission to the sleep laboratory and for 1 week (7 days), the participants wore an actigraph and were instructed to maintain a regular sleep-wake behavior with their usual 7–8 h of sleep (i.e., in bed from 23:00 to 01:00 until 07:00 to 09:00) and to avoid late schedules. The compliance with these recommendations was verified through actigraphic recordings (MW8, CamTech; United Kingdom) that were inspected by the research team at the participant’s arrival the morning before the first night of sleep restriction. During the “sleep restriction” session, the participants were instructed to restrict their sleep time to 3 h for two consecutive nights (i.e., in bed from 03:00 to 06:00; Figure 1A). During the sleep restriction period, the participants continued to follow their usual routine outside the laboratory. After the second sleep-restricted night, the participants returned to the laboratory on the morning of day 3 and their actigraphic recordings were immediately analyzed to ensure their compliance with the imposed sleep-wake hours. During day 1 (after habitual sleep, control condition) and day 3 of each session, the participants remained in the sleep laboratory from 09:00 to 19:00 under continuous supervision and under dim light conditions (luminance level ≤10 lux). In order to help the participants staying awake, from the moment they left the laboratory until their return to the laboratory on day 3 at 09:00, two investigators exchanged text messages with the participants at random times during the entire period outside of the laboratory. Text messages were sent throughout the night (except during the period where participants were instructed to sleep, that is between 3 and 6 a.m.). Participants had to respond right after receiving these messages. In case of an absence of response, participants were immediately called on their personal phone. For lunch, participants received controlled meals consisting of a maximum of 2,500 calories/day with a balanced proportion of nutrients (protein, fat, and carbohydrates). Intake of any medication, alcohol or xanthine derivatives (coffee, tea, chocolate or cola) was prohibited throughout the experimental period. In the “sleep restriction + blue light” session, the same participants repeated the protocol described above, but with the inclusion of four 30-min sessions of blue light exposure (using a Philips^®^ goLITE BLU light) at 10:30, 13:00, 15:30 and 18:00 on the day after the second sleep-restricted night (day 3; Figure 1B). The participants sat facing a table on which was positioned the light device at a distance of approximatively 60 cm. The luminance at the eye level in this seated position was measured using lux meter (Konica Minolta T-10A) and was maintained at 100 lux. The distance to the light source was kept constant across individuals. Participants were instructed not to look directly at the light source. Finally, all assessments were performed post-light administration, and not during the exposure.

### Activity-Based Sleep Monitoring (Actigraphy)

Actigraphy and sleep diaries were used to record compliance with the imposed sleep-wake schedule. During the study period, all subjects were continuously monitored by an actigraphic device (MW8, CamTech; United Kingdom) mounted on a wristwatch and worn on the non-dominant wrist, coupled with a sleep diary that participants filled in to describe their sleep during the study period (Sadeh, 2011). The actigraph was set to sample movements in 1-min periods. Data were recorded *via* a sensor placed within the wrist device, and data analysis (Actiwave MW8 software) assessed the different parameters of the activity/rest cycle. Sleep start and sleep-wake states were assessed independently and as follows: sleep-wake discrimination algorithm is based solely on the presence (or absence) of movement while sleep start is defined using the first immobile block of at least 10 min following bedtime containing no more than one epoch of movement. The sleep diary was used to corroborate the actigraphic data. The following parameters were extracted following routines implemented in the actigraphy software (Oakley, 1997): TST that is the sum of all sleep epochs; bedtime, get-up time and sleep efficiency (Natale et al., 2014). No sleep episodes were detected outside of the scheduled experimental time in bed.

### Assays and Measurements

Saliva samples were collected by passive drool within each session on days 1 and 3 at 11:00, 13:30, 16:00, and 18:30. To avoid any interference with the transient and fast action of alpha-amylase as a digestive enzyme, saliva sampling was performed after at least a 90-min interval following the end of the snack period. Saliva samples were immediately placed on ice and then stored at −80°C until assayed. For alpha-amylase measurements, samples were centrifuged at 3,000 × *g* for 10 min, while cortisol, IgA and testosterone were centrifuged at 1,500 × *g* for 15 min. Samples were assayed for alpha-amylase using a commercial kit (IBL International; Hamburg, Germany), while cortisol, IgA and testosterone were determined using an ELISA kit (Salimetrics; State College, PA, United States). Data for IgA levels were measured relative to saliva flow rate (Obayashi, 2013). Assays were made in duplicate, and the intra- and inter-assay coefficients of variation (CVs) were 3.0 and 3.0% for cortisol, 6.7 and 3.6% for alpha-amylase, 4.50 and 8.65% for IgA, and 2.5 and 5.6% for testosterone, respectively. The analytical ranges of sensitivity for cortisol, alpha-amylase, IgA and testosterone were 0.33–83 nmol.L^–1^, 2–400 U.mL^–1^, 12.5–3,000 μg.ml^–1^, and 3.5–2,080 pmol.L^–1^, respectively.

### Visual Analog Scales, Sleepiness and Mood States

Subjective sleepiness using the Stanford Sleepiness Scale (Hoddes et al., 1973) and visual analog scales for the calm and tense (Monk, 1989) were measured within each session on days 1 and 3 at the same time as the salivary samples at 11:00, 13:30, 16:00 and 18:30. Mood states (tension, depression, anger, vigor, fatigue, confusion, vigor, friendliness, and mood disturbance) were measured at 19:00 using the French version of the Profile of Mood States (POMS; Cayrou et al., 2003).

### Go/NoGo Task

Participants performed a Go/NoGo task within each session on days 1 and 3 at 11:20, 13:50, 16:20, and 18:50. The Go/NoGo task consisted of the presentation of two distinctive stimuli on a computer screen (*N* = 400 presentations for each session). For one stimulus (displayed as the letter P on the screen), volunteers were instructed to press a response button as fast as possible (i.e., the “Go” trials). For the other stimulus (displayed as the letter R on the screen), volunteers had to refrain from responding (i.e., the “NoGo” trials). The proportion of trials was always 80% “Go” trials and 20% “NoGo” trials (*N* = 320 and 80 trials, respectively). Subjects were given a maximum of 2 s to respond, and each response was directly followed by a new trial, forcing the participants to perform the task continuously. The Go/NoGo task is a paradigm frequently used to assess vigilance and sustained attention as it allows to estimate participants’ level of responsiveness (through the examination of Go trials’ accuracy and reaction times) as well as their levels of executive functions (participants’ ability to inhibit automated responses, estimated with the percentage of errors to NoGo trials). To reflect this balance between responsiveness and executive control, we extracted and analyzed the response times on the Go trials (time elapsed between stimulus onset and participants’ response), as well as the percentage of NoGo and Go errors (Sagaspe et al., 2012; Rabat et al., 2016). In order to estimate participants’ overall performance on the task, we computed the d’ using the Signal Detection Theory (Macmillan and Creelman, 2004), which combines the performance on both Go and NoGo trials.

### Memory Task

To examine the influence of sleep restriction and exposure to blue light on long-term cognitive processes, participants performed a memory task. The task was divided into two sequences: an encoding session and several recall sessions. In the encoding session, words were displayed on screen and participants were instructed to determine whether the words displayed were masculine or feminine. The task was done in French, a language in which nouns are either masculine or feminine. During the exposure, a list of 24 words were presented to participants in random order and each word was presented a total of five times (*N* = 120 presentations). 20 lists of 24 bi-syllabic French nouns were created (12 masculine, 12 feminine nouns) and equalized for frequency in the French language using the Lexique 3.0 database (New, 2006). During exposure, one list out of 20 was selected and displayed to participant. Each word was presented until participants indicated their response. This exposure session was performed at 10:45. Following the exposure session and in the same day, four recall sessions were performed at 11:00, 13:45, 16:15, and 18:45 (between the sampling of saliva and the Go/NoGo task). In the recall session, participants were presented with words from the exposure list mixed with new words taken from a new list of masculine and feminine words never presented to participants. Participants were instructed to indicate, for each word, if it belonged or not to the list presented in the morning (classical old/new memory paradigm) (Ebbinghaus, 1885; Eichenbaum et al., 2007; Andrillon and Kouider, 2016). During the recall test, the proportions of novel and old words were identical (*N* = 24, chance level = 50%). For a given participant, the list of new words were presented only once so that new words would never be presented twice to a given participant. Words were displayed on screen until participants delivered their response. The first recall session was performed immediately after the exposure session and can be used as a baseline of memory retention. We computed participant’s performance for each four recall sessions (% of correct responses in classifying words as old or new). For each trial, participants also reported also their confidence in their responses on a scale from 1 (“not sure at all”) to 5 (“absolutely certain”).

### Statistics

The primary endpoint chose before analysis was the effect of blue light on the relative variation of cortisol levels (our previous studies reported variations in baseline cortisol levels between experimental sessions, e.g., Arnal et al., 2016; Rabat et al., 2016). We calculated using our precedent studies that the number of subject needed to observed a significant effect of blue exposure (no changes in cortisol level) compared to habitual effect of sleep restriction (decrease of 30%) with a habitual 15% coefficient of variation and a risk alpha = 0.05 and a power of 0.8 was 12 subjects. In order to mitigate potential data loss (e.g., exclusion of a participant due to the lack of compliance to our study’s requirements), 17 subjects have been recruited and included in the study.

The effects of sleep restriction were evaluated by two-way repeated measures ANOVAs, with a sleep condition factor (habitual sleep and sleep restriction for the “sleep restriction” and “sleep restriction + blue light” sessions) and a time factor, completed by a pairwise comparison *post hoc* test (Student–Newman–Keuls test).

To test the effects of blue light, comparisons were made between the different sleep restriction conditions using normalized delta scores: (restriction–habitual sleep)/habitual sleep for the “sleep restriction” session, and (restriction + blue light–habitual sleep)/habitual sleep) in the “sleep restriction + blue light” session. The normalized changes from habitual sleep were then evaluated by a two-way repeated measures ANOVA with a sleep restriction condition factor (sleep restriction or sleep restriction + blue light) and a time factor, completed by a pairwise comparison *post hoc* test (Student–Newman–Keuls test). The comparisons of delta scores normalized to each session baseline allow to exclude a session effect regarding baseline values.

For the analyses of actigraphy and mood scores, comparisons among the sessions were performed for each sleep parameter using either a paired *t* test or one-way repeated measures ANOVA, completed by a pairwise comparison *post hoc* test (Student–Newman–Keuls test). Data were analyzed using the GraphPadPrism7 software (GraphPad Software^®^). The data displayed a normal distribution (Shapiro–Wilk normality test). Mean values were computed and the group-level variance was expressed using the SEM. A probability level of *P* < 0.05 was considered to be statistically significant.

## Results

### Activity-Based Sleep Monitoring (Actigraphy)

During habitual sleep nights, subjects slept in the “sleep restriction” session for 6 h 57 min ± 54 min (mean ± SEM) on average with a bedtime of 23 h 46 min ± 29 min, a get-up time of 7 h 53 min ± 23 min and a sleep efficiency of 90.1 ± 2.5%; and in the “sleep restriction + blue light” session slept for 7 h 11 min ± 69 min with a bedtime of 23 h 24 min ± 48 min, a get-up time of 7 h 55 min ± 32 min and sleep efficiency of 8.8 ± 3.1%. During the first and second restricted nights, subjects slept 2 h 53 min ± 10 min (bedtime of 2 h 44 min ± 6 min, a get-up time of 6 h 04 min ± 08 min and a sleep efficiency of 93.1 ± 3.3%) and 3 h 06 min ± 11 min (bedtime of 2 h 43 min ± 6 min, a get-up time of 6 h 02 min ± 06 min and a sleep efficiency of 95.1 ± 2.1%) in the “sleep restriction” session, respectively. In the “sleep restriction + blue light” session, during the first and second restricted nights, TST was 2 h 59 min ± 18 min (bedtime of 2 h 44 min ± 4 min, a get-up time of 5 h 57 min ± 09 min and a sleep efficiency of 91,5 ± 4,9%) and 3 h 06 min ± 12 min (bedtime of 2 h 43 min ± 5 min, a get-up time of 6 h 07 min ± 10 min and a sleep efficiency of 93.5 ± 3.8%), respectively. No significant differences were measured in TST, bedtime, wake time and sleep efficiency between the “sleep restriction” and “sleep restriction + blue light” sessions for habitual and sleep restricted nights, indicating good experimental reproducibility prior and during the sleep restriction procedure (all *P* values > 0.05 between the “sleep restriction” and “sleep restriction + blue light” sessions).

### Salivary Cortisol, Testosterone, Alpha-Amylase and IgA After Sleep Restriction Without or With Blue Light

**FIGURE 2:**
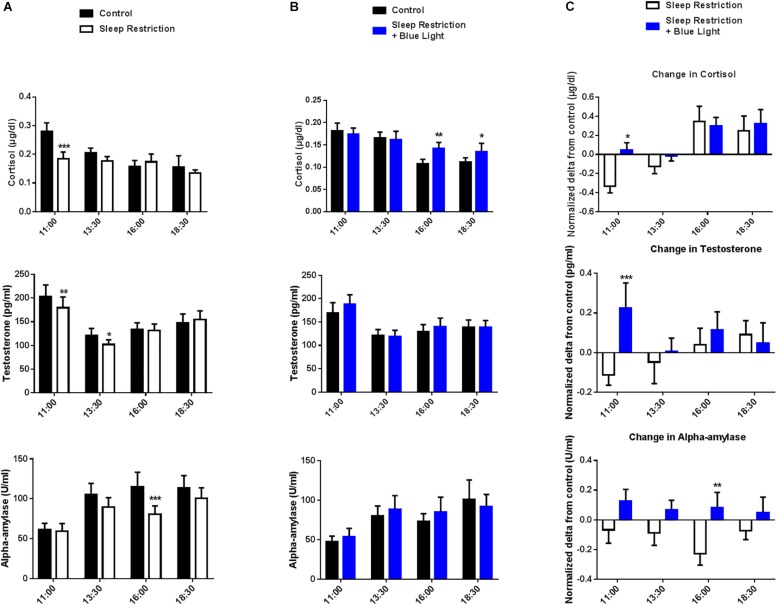
Salivary cortisol, testosterone and alpha-amylase levels were measured every two-and-a-half hours during the daytime period (11:00 to 18:30). Control values after habitual sleep and values after sleep restriction without **(A)** or with blue light exposition **(B)** are shown. To test the effects of blue light, comparisons between the “sleep restriction” and “sleep restriction + blue light” conditions were performed using normalized delta scores **(C)**. Normalized changes from habitual sleep are shown for cortisol, testosterone and alpha-amylase. Mean ± SEM; ^∗^*P* < 0.05, ^∗∗^*P* < 0.01, ^∗∗∗^*P* < 0.001 vs. the respective same time control sleep conditions.

A time effect was observed in both ‘sleep restriction’ and “sleep restriction + blue light” sessions, confirming the circadian influence on cortisol levels (both *P* < 0.01). In the “sleep restriction” sessions, there was a significant interaction (*F*(3,48) = 2.99, *P* = 0.03) between time and sleep condition (habitual sleep vs. sleep restriction) reflecting the fact that sleep restriction did not have the same effect on cortisol level throughout the day with reduced levels in the morning (*post hoc* test: *P* = 0.0009; Figure 2A). When subjects were exposed to blue light (“sleep restriction + blue light”), a significant interaction between time and sleep condition was still observed (*F*(3,45) = 4.03, *P* = 0.03) but there was no longer lower levels of cortisol in the morning (*post hoc* test: *P* = 0.49 at 11:00). Rather, cortisol was increased in the afternoon (*P* = 0.001 at 16:00 and *P* = 0.02 at 18:30; Figure 2B), evidencing a stimulating effect of blue light on cortisol secretion.

We also observed a significant interaction between time and sleep condition for the level of salivary testosterone in the “sleep restriction” sessions (*F*(3,48) = 3.46; *P* = 0.02). *Post hoc* tests revealed a reduction in testosterone levels after the two sleep-restricted nights in the morning and early afternoon as compared to the same period during the control day (*P* = 0.004 and 0.01 at 11:00 and 13:30; Figure 2A). In the “sleep restricted + blue light” sessions, this interaction was no longer significant (*F*(3,45) = 1.7, *P* = 0.17; Figure 2B), evidencing a change in testosterone secretion following exposure to blue light.

In the afternoon following the two sleep-restricted nights and in the “sleep restriction” sessions, we observed again an interaction between sleep condition and time (*F*(3,48) = 2.8, *P* = 0.04), with a decrease in alpha-amylase starting at 13:30 and a clear reduction at 16:30 following sleep restriction (*post hoc* tests: *P* = 0.058 and <0.0001 at 13:30 and 16:00, respectively; Figure 2A). Once again, this interaction was no longer significant in the “sleep restriction + blue light” sessions (*F*(3,45) = 0.85, *P* = 0.47; Figure 2B).

Finally, when assaying salivary IgA, we did not find any significant changes during the day following the two sleep-restricted nights, as compared to the same periods during the control day. There was a time effect, but no significant interaction between time and the sleep condition in the “sleep restriction” session (*F*(3,48) = 13.71, *P* < 0.0001 and *F*(3,48) = 1.171, *P* = 0.33, respectively). Similarly, in the “sleep restricted + blue light” sessions, we observed an effect of time (time effect *F*(3,48) = 7.89, *P* = 0.0003) but no interaction between time and sleep condition (*F*(3,48) = 0.84, *P* = 0.48). suggesting that sleep restriction did not influence the secretion of IgA.

To specifically assess the effect of blue light on these physiological markers, normalized changes from habitual sleep in salivary cortisol, testosterone and alpha-amylase levels during the sleep-restricted day were compared between the “sleep restriction” and the “sleep restriction + blue light” sessions. There was a significant effect of the times of testing for cortisol (*F*(3,45) = 9.69, *P* < 0.0001) with reduced cortisol changes upon sleep deprivation after exposure to blue light (*post hoc* test: *P* = 0.01 at 11:00; Figure 2C). Also, a significant interaction between time and blue-light condition (*F*(3,45) = 3.38, *P* = 0.02) indicated an increase in morning testosterone levels following of blue light exposure (*post hoc* test: *P* = 0.0004 at 11:00; Figure 2C). Finally, there was a main effect of conditions for alpha-amylase (exposure to blue light or not: *F*(1,15) = 6.37, *P* = 0.023) with a significant difference in the afternoon for the blue light condition (*post hoc* test: *P* = 0.005 at 16:00; Figure 2C). In sum, these results indicate an enhancing effect of blue light on cortisol and testosterone in the morning and alpha-amylase in the afternoon following sleep restriction.

### Go/NoGo Task: Testing Executive Performance of Inhibitory Motor Control After Sleep Restriction Without or With Blue Light

**FIGURE 3:**
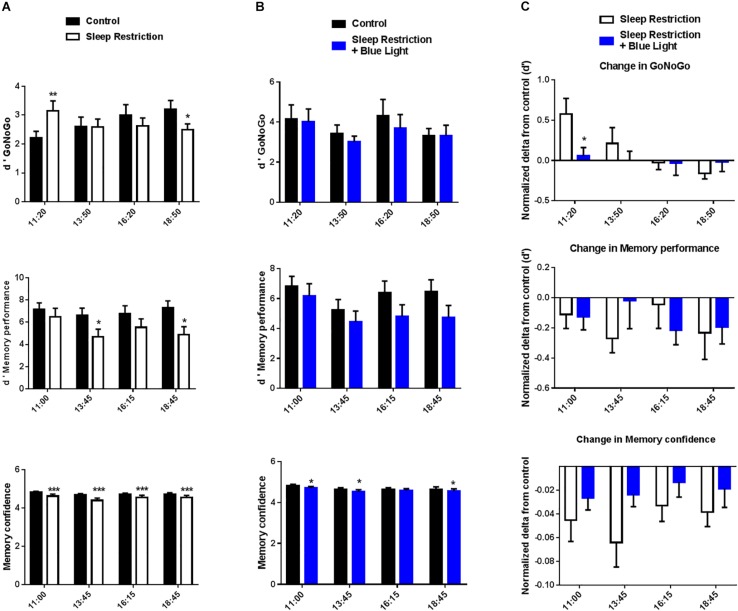
d’ GoNoGo, d’ memory performance, and memory confidence ratings were repeatedly measured during the daytime period (11:00 to 19:00). Participants’ performance in the GoNoGo task was calculated by using the d’, which takes into account both errors on Go and NoGo trials. For the memory, we focused here on the recall performance. d’ memory performance quantifies participants’ ability to discriminate between new and old items. Participants also reported their confidence in their recall responses to capture implicit forms of memory. Control values after habitual sleep and values after sleep restriction without **(A)** or with blue light exposition **(B)** are shown. To test the effects of blue light, comparisons between the “sleep restriction” and “sleep restriction + blue light” conditions were performed using normalized delta scores **(C)**. Normalized changes from habitual sleep are shown for d’ GoNoGo, d’ memory performance, and memory confidence. Mean ± SEM; ^∗^*P* < 0.05, ^∗∗^*P* < 0.01, ^∗∗∗^*P* < 0.001 vs. the respective same time control sleep conditions.

Participants’ performance was calculated by using the d’ (see section “Materials and Methods”). Performance changed throughout the day following the two nights of sleep restriction and in the absence of blue light (“sleep restriction” sessions), as evidenced by a significant interaction between time and sleep condition (*F*(3,45) = 9.49, *P* < 0.001) (Figure 3A). There was however no main effect of sleep restriction (*F*(1,15) = 0.02, *P* = 0.88), suggesting the fact that sleep deprivation did not have the same impact on performance throughout the day. Indeed, we observed, paradoxically, an increase in performance in the morning following sleep restriction (11:00, *P* = 0.0098, *post hoc* test), followed by a decrease in performance in the afternoon (18:00, *P* = 0.0185, *post hoc* test). Interestingly, in the “sleep restriction + blue light” sessions, there was no main effect of sleep restriction on performance (*F*(1,15) = 0.66, *P* < 0.43) and no interaction between sleep restriction and time of the day (*F*(3,45) = 0.45, *P* = 0.71) (Figure 3B). Accordingly, none of the times of testing led to significant differences when comparing before and after sleep restriction (all *P* > 0.05, *post hoc* tests).

The interaction for the “sleep restriction” sessions and its absence in the “sleep restriction + blue light” was driven by the performance on NoGo trials (i.e., NoGo errors), which show a similar pattern of results: an interaction between time and sleep condition in “sleep restriction” sessions (*F*(3,45) = 8.94, *P* < 0.001) and no interaction in “sleep restriction and blue light” sessions (*F*(3,45) = 1.31, *P* = 0.28). A similar but weaker interaction was observed for response-times, another indicator of performance, in the “sleep restriction” sessions (*F*(3,45) = 3.18, *P* = 0.033). Once again, the interaction between time and sleep condition was not significant for the “sleep restriction + blue light” sessions (*F*(3,45) = 1.79, *P* = 0.16). Overall, it seems that the exposure to blue light leads to a stabilization of performance and executive control, with an absence of observable decline in performance from morning to evening, following sleep restriction (no time x sleep condition interactions).

When comparing sessions with or without blue light exposure, the main effect of exposure to blue light on Go/NoGo performance changes (d’) was not significant (*F*(1,15) = 1.6, *P* = 0.23) but there was a significant interaction between the exposure to blue light and the timing of the tests (*F*(3,45) = 2.98, *P* = 0.041). It appeared the exposure to blue light tended to stabilize participant’s performance throughout the day in contrast with the sleep-restriction only condition in which participants were better in the beginning of the day but worse toward the end (Figure 3C). Accordingly, *post hoc t*-tests revealed significant differences in the d-prime change between exposure or non-exposure to blue light in the morning (11.00, *P* = 0.03). However, this effect was in favor of the session without blue light. Exposure to blue light led to a trend for better performance toward the end of the day (18.30) but this difference did not cross significance (*P* = 0.14).

### Memory Task: Testing Higher-Order Cognitive Abilities After Sleep Restriction With or Without Blue Light

For the memory task, we analyzed the recall performance throughout the day (i.e., were participants able to remember which items had been presented in the morning and which item were new). Once again, we extracted a d-prime to quantify participants’ ability to discriminate between new and old items. Memory performance on the recall test decreased after sleep deprivation in the “sleep restrictions” session (*F*(1,15) = 7.4, *P* = 0.016), evidencing the impact of sleep debt on memory performance (Figure 3A). *Post hoc t*-tests confirm a decrease in recall performance between the baseline day and after sleep restriction in the afternoon at 13.45 and 18.45 (*post hoc t*-tests, *P* = 0.015 and 0.010, respectively). When participants were exposed to blue light, the effect of sleep restriction on memory performance was marginal (*F*(1,15) = 4.2, *P* = 0.058) although we observed an effect of the timing of the memory test (*F*(3,45) = 3.3, *P* = 0.03) in these “sleep restriction + blue light” sessions (Figure 3B). Similarly as in the Go/NoGo test, these results suggest a stabilization of performance following exposure to blue light and after sleep restriction.

Without exposure to blue light, there was a significant effect of sleep restriction on participants’ confidence as well (*F*(1,15) = 9.8, *P* = 0.007) (Figure 3A). *Post hoc t*-tests revealed that, for all memory tests performed after sleep restriction and without exposure to blue light, participants reported less confidence in their response (all *P* < 0.0001, *post hoc t*-tests). There was also a significant interaction between sleep restriction and timing of the day (*F*(3,45) = 2.9, *P* = 0.045). When sleep restriction was accompanied by the exposure to blue light, the effect of sleep restriction on confidence was still significant (*F*(1,15) = 5.4, *P* = 0.034) (Figure 3B). *Post hoc t*-tests revealed differences in the first part of the day (11.15 and 13.45) and the last part of the day (18.45) between baseline and after sleep restriction (*P* = 0.02, 0.03, and 0.02, respectively). However, there was no longer a significant interaction between sleep restriction and timing of the day (*F*(3,45) = 0.48, *P* = 0.70).

However, the stabilizing effect of the blue light reported for Go/NoGo performance was not observed in the memory task when looking at changes in performance (main effect of exposure to blue light *F*(1,15) = 0.12, *P* = 0.74, interaction with timing of the day: *F*(3,45) = 1.55, *P* = 0.21) or confidence (main effect of exposure to blue light *F*(1,15) = 1.84, *P* = 0.20; interaction with timing of the day: *F*(3,45) = 0.91, *P* = 0.44) (Figure 3C). This pattern of results suggests that the impact of blue light on cognition might be task-specific (see Discussion).

### Subjective Sleepiness and Visual Analog Scales of Stress and Mood States After Sleep Restriction Without or With Blue Light

**FIGURE 4:**
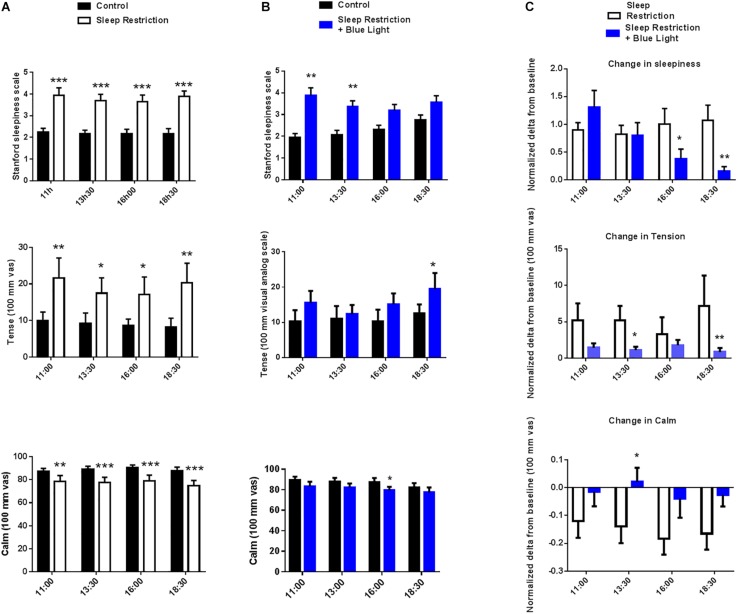
Stanford sleepiness scales, tense and calm visual analog scales, were measured every two-and-a-half hours during the waking period (11:00 to 18:30). Control values after habitual sleep and values after sleep restriction without **(A)** or with blue light exposition **(B)** are shown. To test the effects of blue light exposure, comparisons between the “sleep restriction” and “sleep restriction + blue light” conditions were performed using normalized delta scores **(C)**. Normalized changes from habitual sleep are shown for Stanford sleepiness scales, tense and calm visual analog scales. Mean ± SEM; ^∗^*P* < 0.05, ^∗∗^*P* < 0.01, ^∗∗∗^*P* < 0.001 vs. the respective same time control sleep conditions.

In sleep-restricted participants Stanford Sleepiness scale scores were around twofold higher at all tested time points (main effect of sleep restriction *F*(1,16) = 44.35, *P* < 0.0001; *post hoc* test: *P* < 0.0001 at 11:00, 13:30, 16:00, and 18:30 vs. the same time control condition; Figure 4A). After exposure to blue light, there was still a main effect of sleep restriction (*F*(1,16) = 9.707, *P* = 0.007) and a time effect (*F*(3,48) = 2.83, *P* = 0.04). The sleepiness score was still higher in the morning at 11:00 and 13:30 (*post hoc* test: *P* = 0.007 and *P* = 0.004) but, with the repetition of blue light exposure throughout the day, participants progressively felt less sleepy and, toward the end of the day, there was no longer any significant difference between the condition with sleep restriction and the habitual sleep condition (*P* = 0.18 and *P* = 0.13 at 16:00 and 18:30 vs. the same time control condition, respectively; *post hoc* test; Figure 4B).

Visual analog scales for the tense and calm subscales were assessed at 11:00, 13:30, 16:00, and 18:30. In the ‘sleep restriction only’ sessions, a marked rise in the perceived tense feeling (main effect of sleep restriction *F*(1,16) = 6.54, *P* = 0.021) was recorded at the same time points tested following sleep restriction (*P* = 0.002, *P* = 0.03, *P* = 0.02, and *P* = 0.002 at 11:00, 13:30, 16:00, and 18:30 vs. the same time control condition, respectively; *post hoc* test; Figure 4A). In the same sessions, all time points also displayed a reduction in the perceived feeling of calmness following sleep restriction (main effect of sleep restriction *F*(1,16) = 10.2, *P* = 0.005; *post hoc t*-tests: *P* = 0.007, *P* = 0.0006, *P* = 0.0005, and *P* = 0.0001 at 11:00, 13:30, 16:00, and 18:30 vs. the same time control conditions, respectively; Figure 4A). When participants were subsequently exposed to blue light, there was still a significant effect of sleep condition (*F*(1,16) = 7.1, *P* = 0.017), but *post hoc* tests revealed that the level of the perceived tense feeling was no longer increased following sleep restriction except for the last time point in the afternoon at 18:30 (*P* = 0.0986, *P* = 0.6753, *P* = 0.1197, and *P* = 0.0295 at 11:00, 13:30, 16:00, and 18:30 vs. the same time control condition, respectively; *post hoc* test; Figure 4B). Similarly, in the “sleep restriction + blue light sessions,” there was still a main effect of sleep condition on the perceived feeling of calmness (*F*(1,16) = 5.21, *P* = 0.03) but *post hoc t*-tests showed that the level of calmness no longer differed from the baseline day, except at 16:00 (*P* = 0.06, *P* = 0.08, *P* = 0.01, and *P* = 0.12 at 11:00, 13:30, 16:00, and 18:30 vs. the same time control condition, respectively; *post hoc* test; Figure 4B). No time effect or interaction between time and the sleep condition was significant for the calm or tense scales.

The effects of sleep restriction on sleepiness depended on the exposure to blue light (main effect of blue light: *F*(1,16) = 2.8, *P* = 0.04). The alerting effects of light became significant in the middle and the end of the afternoon (*post hoc* tests: *P* = 0.04 and *P* = 0.0038 at 16:00 and 18:30, respectively; Figure 4C). Blue light also significantly reduced participants’ perceived tense and calm ratings (main blue light effect *F*(1,16) = 0.53, *P* = 0.04 and *F*(1,16) = 5.23, *P* = 0.03) compared to sleep restriction without blue light. The stress-releasing effects of light were measured during the afternoon (13:30 and 18:30 for tense EVA; *P* = 0.034 and *P* = 0.001, respectively, and 13:30 for calm EVA *P* = 0.04; *post hoc* test; Figure 4C).

**FIGURE 5:**
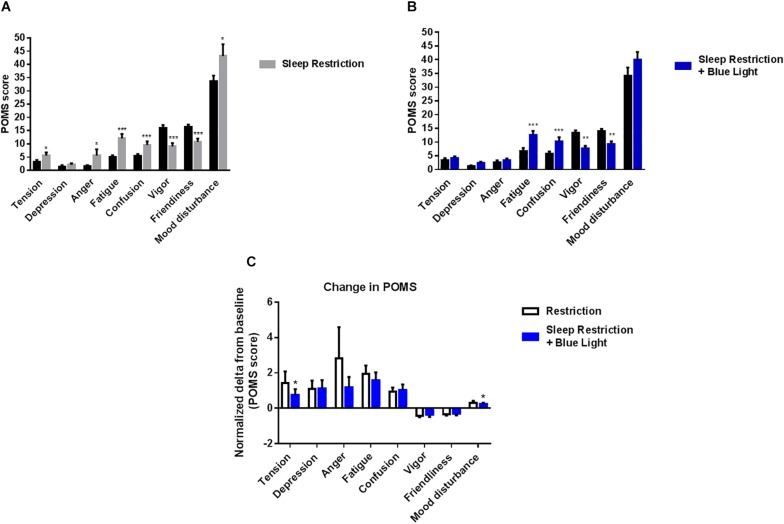
Profile of mood states were measured at the end of the afternoon (19:00). Control values after habitual sleep and values after sleep restriction without **(A)** or with blue light exposition **(B)** are shown. To test the effects of blue light, comparisons between the “sleep restriction” and “sleep restriction + blue light” conditions were performed using normalized delta scores **(C)**. Normalized changes from habitual sleep are shown for profile of mood states. Mean ± SEM; ^∗^*P* < 0.05, ^∗∗^*P* < 0.01, ^∗∗∗^*P* < 0.001 vs. the respective same time control sleep conditions.

Finally, participants’ mood was assessed once per day at the end of afternoon. Tension, anger, fatigue, confusion and mood disturbance scores were all increased after sleep restriction (*P* = 0.03; 0.04; 0.0002; <0.0001; and 0.03, respectively). In the same time, vigor and friendliness scores were reduced following the sleep-restricted period (*P* < 0.0001 for both respectively; Figure 5A). When the participants were punctually exposed to blue light during the day while being sleep deprived, tension, anger and mood disturbance scores were not significantly different from pre-restriction values (all *P* > 0.1) but fatigue, confusion, vigor, and friendliness were similarly affected as in the sessions without blue light exposure (all *P* < 0.01) (Figure 5B). The effects of blue light on the mood state scores were significantly measured for the tension and mood disturbances components (*P* = 0.04 and 0.03, respectively; Figure 5C).

## Discussion

We report here that blue light exposure (lasting no longer than 30 min at a time) after two nights of restricted sleep (3 h) restores several biological and behavioral markers affected by sleep restriction. Exposure to blue light prevented sleep restriction-related decreases in morning cortisol and testosterone and afternoon alpha-amylase, as well as the increases in subjective stress, sleepiness and deteriorated executive control (Go/NoGo task). Importantly, exposure to blue light stabilized cognitive performance toward their baseline levels in a simple attention task but did not improve memory retention, pointing toward a task-specific effect on cognitive abilities.

### Blunted Response of Stress and Gonadal Systems in Our Semi-Ecological Sleep Restriction Design

In order to closely approximate everyday life, we designed and implemented a semi-ecological protocol, which could have several advantages. First, participants slept in their own home, minimizing the impact of sleeping in an unfamiliar environment. Second, although biological samplings and tests were performed in the laboratory, outside of these periods the participants maintained their normal routine, including out-of-home activities that could potentially activate more stress systems than if they had remained in the laboratory for the duration of the experiment. Indeed, in a similar study, participants remained in the laboratory where no unexpected events could challenge them. Despite using identical stress scales and a similar sleep restriction protocol (two nights of 4-h sleep between 01:00 and 05:00) as in our study, there was no increase in subjective stress contrary to what we report here (Guyon et al., 2014). This corroborates the idea that coping with everyday life stressors (as in our design) could potentiate the impact of sleep restriction by activating the stress system at a higher level than when participants remain entirely in the laboratory, where environmental stressors are typically kept at a minimal level.

Importantly, our semi-ecological protocol replicated the cortisol and alpha-amylase expression profiles during the baseline day which are consistent with the literature. Specifically, after habitual sleep, the highest salivary cortisol values were measured in the morning, whereas the highest salivary alpha-amylase values were measured in the afternoon, as previously reported in healthy participants of the same age (Van Lenten and Doane, 2016). Predominant morning HPA activity relayed by SNS activity in the afternoon could explain these temporal profiles. Importantly, following sleep restriction, cortisol was decreased in the morning and alpha-amylase in the afternoon, which could be mediated by a negative feedback of the stress system on morning HPA and afternoon SNS. That is, the recruitment of the stress system by extended awakening could have led to morning HPA and afternoon SNS hyporeactivity and a blunted cortisol and alpha-amylase response. Interestingly, Guyon et al. (2014) reported a circadian dampening (−20%) of daytime serum cortisol amplitude after two nights of sleep restricted to 4 h but did not report any change in serum or associated morning salivary cortisol, in link with the absence of any measured psychological stress.

However, our results are very similar to those obtain after a longer period of laboratory-based sleep restriction [i.e., 7 days (Rabat et al., 2016)]. In this study, participants were sleep deprived to 4-h of sleep per night and for a period of 7 days (in bed from 02:00 to 06:00). Similarly in our study, lower cortisol and alpha-amylase morning levels were measured at the end of the sleep restriction period. These reductions in cortisol and alpha-amylase were also linked to lower objective and subjective alertness and increased NoGo response errors.

We also observed a reduction in the level of morning testosterone following sleep restriction. This androgen deficiency could participate in the lower vigor and alertness reported here. Accordingly, previous studies have reported a decrease in testosterone levels after robust sleep deprivation (one night of total sleep deprivation or eight nights of 5-h sleep) in humans (Leproult and Van Cauter, 2011; Arnal et al., 2016). Thus, our data suggest that event short protocols of sleep restriction (2 days of 3-h long restricted sleep) could produce results obtained after a longer period of laboratory-based sleep restriction when using a semi-ecological protocol.

### Bi-Phasic Response of Stress and Gonadal Systems and Cognitive Performance in Sleep-Deprived Subjects

Our results revealed a biphasic response, after sleep restriction, with an initial decrease in the levels of cortisol and testosterone followed by a gradual increase throughout the day. Interestingly, this bi-phasic response of markers of physiological stress was mirrored by a bi-phasic modulation of cognitive performance on a Go/NoGo task (see “Sleep restriction condition,” Figure 2C cortisol and testosterone and Figure 3C change in GoNoGo). Indeed, following sleep restriction, performance increased in the morning when comparing to the pre-restriction. Then performance decreased throughout the day. This initial increase is unlikely to be solely due to training as the effect was not present when participants were exposed to blue light. It is therefore possible that the bi-phasic response of physiological markers of stress and of cognitive performance evidence the combined action of short-lived compensatory mechanisms which cannot prevent a decrease in performance later during the day.

### Cortisol, Alpha-Amylase, Testosterone, Alertness and Cognitive Performance Responses to Light Exposure

Previous data have more specifically indicated that after a typical night, 1 h of white light exposure (800 lux) 20–40 min after morning awakening can enhance salivary cortisol expression, although no change was measured in the evening during a similar exposure (Scheer and Buijs, 1999). We identified a beneficial effect of blue light exposure on subjective sleepiness associated with significant cortisol increases at 16:00 or 18:30 as compared to sleep restriction alone. The repetitive exposure to blue light also appeared to cancel the bi-phasic modulation of behavioral performance observed in the sleep-restriction condition. With blue light exposure, performances on the Go/NoGo task were stable in time and did not differ between pre and post sleep restrictions. Importantly, this stabilizing effect of cognitive performance was observed only in the Go/NoGo task and not in the memory task. Our results therefore suggest that the repetitive exposure to blue light could cancel the effect of sleep restriction and decrease performance variability throughout the day but these effects might be restricted to certain cognitive functions, potentially to “here and now” performance rather than long-term cognitive abilities (such as memory retention).

These results contrast with other studies using light intervention showing that the administration of white light between 12:00 and 16:00 (5,000 lux) or between 13:00 and 16:00 (2,000–4,000 lux) after a single night of laboratory-based sleep deprivation could not modify salivary or plasma cortisol levels during early afternoon (Leproult et al., 2001; Rüger et al., 2006). However, a mice study indicates that light could stimulate corticosterone release during the subjective night, as well as during the subjective day. This suggests that retinohypothalamic signals may be relayed to the adrenal gland during the subjective day, by cellular mechanisms linked to descending autonomic pathways that are independent of an effect on the clock in the suprachiasmatic nucleus (Kiessling et al., 2014).

In addition, continuous exposure to blue-enriched light for 2 or 5 h in the evening decreased subjective sleepiness and reaction times during sustained attention tasks, i.e., Go/NoGo and psychomotor vigilance tasks in non-sleep deprived healthy young adults with reduced melatonin levels (Cajochen et al., 2011; Chellappa et al., 2011). Nevertheless, these improvements in sustained attention were measured *during* long periods of blue light and not *after* the period of light administration as reported here for a better alertness and accuracy in NoGo responses.

Regarding alpha-amylase and testosterone, to our knowledge, only two studies investigated the effects of daytime light (red and white light) under regular sleep/wake schedule on salivary alpha-amylase or of 2 h of bright light (2,000 lux from 05:00 to 07:00) after chronic sleep restriction on testosterone. Both studies found no effects for both markers (Touitou et al., 1992; Sahin et al., 2014). Considering now the protective role in mucosal immunity of alpha-amylase and the beneficial testosterone effect on the maintenance of male reproductive system and muscle, the stimulating effects of blue light reported here on these molecules deserve further investigations.

### Psychobiological Stress and Cognitive Degradation Resorptions After Short Periods of Blue Light Exposure: Potential Involvement and Applications of a Persistent Effect

We identified a stimulating and persistent effect of repeated short blue light periods acting concomitantly on sustained attention and stress and androgen markers. This short-duration period of blue light is expected to be less aggressive for the retina than longer continuous periods of exposure (>6 h) reported to display potential deleterious effects in rodents (Jaadane et al., 2017). Besides, the latter finding might not be relevant since rats are nocturnal and do not have a macula (which protects this region of the retina in humans).

The continuous exposure to blue-enriched white light in the workplace and during daytime office hours has been reported to improve self-reported alertness, performance and sleep quality (Viola et al., 2018). In view of our results, the use of daytime intermittent blue light exposure in occupational settings could prevent cognitive degradations and bouts of sleepiness and should be further investigated.

## Limitations of the Study

Our study has several limitations which are important to address. First, for the light intervention, we chose to measure parameters after, but not during the periods of light exposure. This was done to make sure that we would examine the impact of light exposure on the circadian clock rather than changes related to the lighting condition itself. Accordingly, in our design, all tests were performed under the same conditions. In addition, the impact of blue light on circadian circuits is expected to extend in time beyond the presentation of blue light. However, this means we could not determine at which time the light effects began to act on our parameters of interest. Furthermore, by comparing blue light to dim light, our design cannot demonstrate the spectral specificity of our intervention (i.e., the fact that it would work specifically with blue light but not with lights with different spectral composition at the same luminance). However, the specificity of the sensitivity of circadian circuits to blue light has been extensively studied in the past making it extremely unlikely that our results would be replicated when using blue-depleted light. Also, the light exposition (dim light) during the experimental days in the lab was not ecological (i.e., different from outdoor or usual artificial light levels). This was done in order to better evidence the impact of blue light but prevents us from concluding on the impact of blue light compared to usual levels of natural or artificial lights.

Second, as we opted for a semi-ecological setting, participants were not sleep deprived under controlled laboratory conditions. Nonetheless, the efficiency of our sleep restriction procedure was confirmed by the continuous actimetry recordings, although we are unable to determine the exact sleep architecture of the restricted nights. Furthermore, since we performed a semi-ecological study (albeit supervised by two investigators through text messages and phone calls for assuring compliance with sleep-wake schedules), we could not completely control the sleep and behavior of volunteers outside of the laboratory (e.g., caffeine consumption). However, no significant differences were measured prior to the two experimental sessions in bedtime or wake time of the participants suggesting that participants behavior and compliance with the instructions were similar across conditions.

Third, to extract information about the physiological response to sleep deprivation and/or blue light, we chose to sample saliva rather than perform multiple blood samplings. This was done in order to avoid the potential stress generated by the blood drawing procedure.

Fourth, we recruited only young males in this study since absolute values of saliva testosterone during daytime are different in men and women (higher concentrations in men) due to a distinct metabolism in salivary glands of men and women (Hofman, 2001). Our results would need to be replicated in more diverse populations including females and/or older adults and younger children.

## Data Availability Statement

All datasets generated for this study are included in the article.

## Ethics Statement

The studies involving human participants were reviewed and approved by Hôtel-Dieu Hospital Ethics Committee (CPP Ile de France 1 – N° 2014-sept.-13690). The patients/participants provided their written informed consent to participate in this study.

## Author Contributions

BF, TA, MC, and DL conceived and designed the study. BF, CG, and AD acquired the data. BF, TA, CD, AS, PV, and MG analyzed the data. BF, TA, DG-M, FS, and DL interpreted the data and wrote the manuscript.

## Conflict of Interest

The authors declare that the research was conducted in the absence of any commercial or financial relationships that could be construed as a potential conflict of interest.
